# Hyperglycemia in the early stages of type 1 diabetes accelerates gastric emptying through increased networks of interstitial cells of Cajal

**DOI:** 10.1371/journal.pone.0222961

**Published:** 2019-10-09

**Authors:** Kazuhisa Kishi, Noriyuki Kaji, Tamaki Kurosawa, Satoshi Aikiyo, Masatoshi Hori

**Affiliations:** Department of Veterinary Pharmacology, Graduate School of Agriculture and Life Sciences, The University of Tokyo, 1–1–1 Yayoi, Bunkyo-ku, Tokyo, Japan; Max Delbruck Centrum fur Molekulare Medizin Berlin Buch, GERMANY

## Abstract

Gastric emptying (GE) can be either delayed or accelerated in diabetes mellitus (DM). However, most research has focused on delayed GE mediated by a chronic hyperglycemic condition in DM. As such, the function of GE in the early stages of DM is not well understood. Interstitial cells of Cajal (ICC) are pacemaker cells in the gastrointestinal tract. In the present study, we investigated changes in GE and ICC networks in the early stages of DM using a streptozotocin-induced type 1 diabetic mouse model. The changes in GE were measured by the ^13^C-octanoic acid breath test. ICC networks were immunohistochemically detected by an antibody for c-Kit, a specific marker for ICC. Our results showed that GE in type 1 DM was significantly accelerated in the early stages of DM (2–4 weeks after onset). In addition, acute normalization of blood glucose levels by a single administration of insulin did not recover normal GE. ICC networks of the gastric antrum were significantly increased in DM and were not affected by the acute normalization of blood glucose. In conclusion, our results suggest that GE is accelerated in the early stages of DM, and it is associated with increased ICC networks. This mechanism may help to clarify a link between the onset of DM and GE disorders.

## Introduction

Diabetes mellitus (DM) is a chronic disease with rapidly increasing prevalence worldwide. Hyperglycemia in DM injures many organs, such as the circulatory organ, kidneys, and nerves. Gastrointestinal (GI) motility is also affected by hyperglycemia in DM. The most common GI disorder in DM is gastroparesis, which is a chronic condition characterized by delayed gastric emptying (GE) without mechanical obstruction [1]. On the other hand, accelerated GE also occurs in patients with type 1 or type 2 [2,3]. Abnormal GE worsens the control of blood glucose levels and increases the risk of DM complications.

Interstitial cells of Cajal (ICC) are interstitial cells in the GI tract. ICC of the myenteric plexus region (ICC-MY) serve as a pacemaker for GI motility [4]. ICC generate spontaneous and rhythmic slow waves that propagate thorough the networks of ICC and smooth muscle cells (SMCs) via gap junctions and promote the spontaneous contractions of smooth muscles [5]. In addition to the regulation of GI function by the enteric nervous system (ENS), the crosstalk between ICC is necessary to produce normal GI motility. Recently, some reports have suggested that the delayed GE seen in patients and rodents with chronic DM is associated with ICC depletion [6,7]. Thus, the disruption in the homeostasis of ICC is considered to be strongly associated with abnormal GE in DM.

There are many reports about GE and the pathological changes in ICC during the chronic stages of DM. In contrast, the changes in GE and ICC in the early stages of DM are not well understood. Understanding the changes in GE and ICC in the early stages of DM is an important prerequisite for maintaining strict control of blood glucose levels and for developing prevention or treatment methods for GE disorders, which, thus far, has not been achieved. In this study, we investigated the changes in GE and the condition of ICC networks in the early stage of DM using a mouse model of type 1 DM.

## Materials and methods

### Animals

All animal care and experimental procedures complied with the Guide for Animal Use and Care published by the University of Tokyo and were approved by the Institutional Review Board of the University of Tokyo (P18-131). Male C57BL/6J mice (10–12 weeks) were used in this study. Animals were housed in sterilized cages with 2–4 mice per 330 cm^2^ and given ad libitum access to water and standard mouse chow. Mice were kept at 22 ± 2°C on a 12 h light/dark cycle. Three times a week, we monitored overall health, movement, fur condition, body weight stability, and access to both food and water of the mice. We frequently replenished water and food and carried out cage cleaning because of polydipsia in diabetic mice. Body weight and blood glucose levels were monitored weekly. In this study, we set an endpoint in the induction of DM. If, after induction of DM, the weight loss rate exceeded 20% in a few days, the mice were euthanized at that time. All animals were euthanized by cervical dislocation under deep isoflurane.

### Experimental models

Streptozotocin (STZ) was used to prepare the diabetic mouse model. STZ is a nitrosourea analogue that causes insulin-dependent DM [8]. STZ (FUJIFILM Wako Pure Chemical Co., Ltd., Tokyo, Japan) was intraperitoneally (i.p) injected at 200 mg/kg of body weight dissolved in an ice-cold 0.1 M citrate buffer. Control mice received an equal volume of citrate buffer by i.p. injection. Blood glucose levels were measured with LabAssay^™^ Glucose (FUJIFILM Wako Pure Chemical Co., Ltd.). One week after the STZ injection, DM was confirmed by the presence of hyperglycemia, and only mice with blood glucose levels >16.7 mmol/l were included in this study. Mice that did not become DM and mice weighing < 20 g at 10–12 weeks old were not used. We used insulin (Lantus insulin glargine, Sanofi-Aventis, Bridgewater, NJ) to temporarily decrease blood glucose levels and maintain glucose levels between 100 and 200 mg/dl. Insulin at a dose of 1.5 U/kg was subcutaneously injected 3 hours before measuring blood glucose levels. The dose of STZ and insulin was determined based on a previous report [9,10].

### Evaluation of gastric emptying

Gastric emptying was evaluated using a ^13^C-octanoic acid breath test, as previously reported [11,12]. The animals were fasted for 8–16 h and placed in a chamber that was large enough for the mice to move freely. After the administration of 200 mg of a test meal consisting of heated egg yolk and 0.2 μl ^13^C-octanoic acid (Cambridge Isotope Laboratories, Inc., MA, U.S.A.) a blow pump device (Thermo Fisher Scientific Inc., Tokyo, Japan) collected breath samples were accumulated in the chamber at a flow rate of 70 ml/min for a duration of 1 min, which were then directed into a breath collection bag (Otsuka Pharmaceutical). Breath samples were collected every 10 min for 120 min, after which breath collection occurred at 140, 160, 180, 210 and 240 min after administration of the test meal.

### Data analysis for the ^13^C-octanoic acid breath test

The ^13^CO_2_/^12^CO_2_ ratio in the breath samples was analyzed using an infrared spectroscopic analyzer (Otsuka Electronics Co., Ltd., Osaka, Japan), and changes in ^13^CO_2_ (Δ^13^C, ‰) were calculated from the ^13^CO_2_/^12^CO_2_ ratio. A mixed gas composed of 5% ^12^CO_2_ and 95% O_2_ was used as a standard. The maximum concentration (C_max_; ‰), the time to reach maximum concentration (T_max_; min) and the area under the exhalation concentration-time curve (AUC_240min_; ‰·min) were calculated using the value of Δ^13^C. The half-life (T_1/2_; min) was calculated from the slope of the elimination phase in the Δ^13^C curve [13–15].

### Whole-mount gastric muscularis preparation

Immunohistochemical analyses were performed using whole-mount gastric muscularis preparations. We used the gastric antrum in this experiment. Whole-mount muscularis samples were prepared as reported previously, with muscularis tissue sheets pinned to the silicon base of dishes [9,16,17]. After fixation in ice-cold acetone for 10 min, the tissue samples were washed with Tris-buffered saline (TBS).

### Immunofluorescence

After fixation and washing, the samples were incubated with TBS containing 2% bovine serum albumin for 1.5 h at room temperature to reduce nonspecific binding of antibodies. This incubation was followed by incubation with rat anti-c-Kit monoclonal antibody (1:500, clone; ACK2, Panapharm Laboratories, Kumamoto, Japan) and rabbit anti-PGP9.5 polyclonal antibody (UltraClone Limited, Isle of Wight, UK) at 4°C overnight. The immunoreactivity was detected using an Alexa Fluor-labeled secondary antibody (1:500, Life Technologies, Gaithersburg, MD, USA). After being washed with TBS, the samples were imaged with a laser-scanning confocal microscope (EZ-C1, Nikon, Tokyo, Japan). The average density of the c-Kit-positive area was calculated using binarized images of 3 randomly selected fields in each sample.

### Determination of oxidative stress

Oxidative stress was determined by measuring serum thiobarbituric acid reactive substances (TBARS) as malondialdehyde (MDA), a product of polyunsaturated fatty acid peroxidation, using TBARS assay (TBARS Assay Kit, Cayman Chemical Company, Michigan, USA). Procedure was performed according to the manufacturer's manual.

### Statistical analyses

All results were expressed as the mean ± standard error (SEM). The data were analyzed using an unpaired Student’s t-test for comparisons between two groups, a one-way analysis of variance (ANOVA), and Tukey’s test for comparisons among time-series data. A value of P < 0.05 was regarded as significantly different.

## Results

### Characterization of the STZ-induced type 1 diabetic mouse model

**Fig 1:**
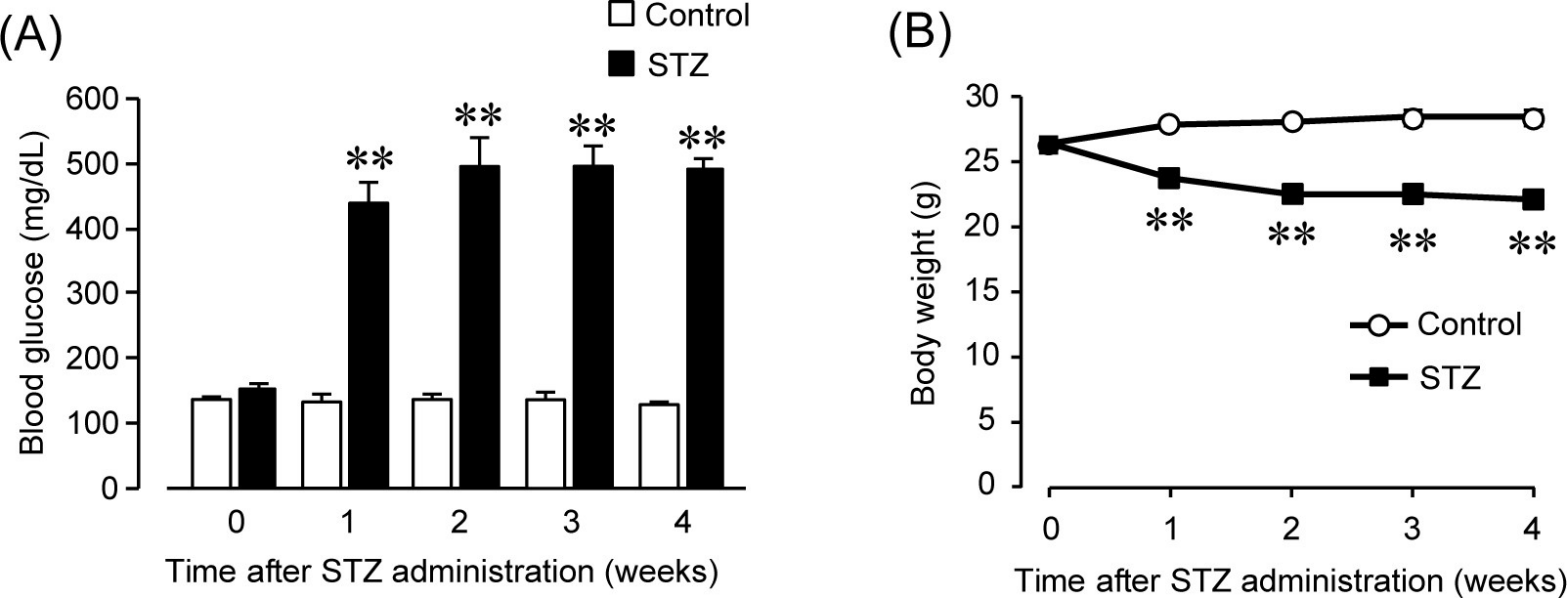
Characterization of the streptozotocin-induced diabetic mouse model. (A) Blood glucose level after an injection of streptozotocin (STZ) in control and diabetic (DM) mice. Each column shows the mean ± SEM (n = 5). **P < 0.01; significantly different from control. (B) Body weight after injection of STZ in control and DM mice. Each column shows the mean ± SEM (n = 5). **P < 0.01; significantly different from control.

We first measured blood glucose levels and body weight to confirm the onset of type 1 DM. Fig 1A shows blood glucose levels at 0, 1, 2, 3 and 4 weeks after the administration of STZ (Fig 1A). The STZ treatment resulted in a 3-4-fold increase in the blood glucose levels at 1, 2, 3 and 4 weeks (control 0 week: 134.9 ± 4.0 mg/dl, 1 week: 132.0 ± 10.0 mg/dl, 2 weeks: 136.1 ± 7.5 mg/dl, 3 weeks: 141.8 ± 9.8 mg/dl, 4 weeks: 127.4 ± 3.4 mg/dl; STZ 0 week: 153.6 ± 6.0 mg/dl, 1 week: 509.3 ± 12.3 mg/dl, 2 weeks: 484.4 ± 43.6 mg/dl, 3 weeks: 493.7 ± 34.3 mg/dl, 4 weeks: 502.1 ± 11.5 mg/dl; < 0.01 control vs. STZ, n = 5). Fig 1B shows the change in body weight after the administration of STZ (Fig 1B). The STZ treatment resulted in a significant weight loss 1 week after the administration of STZ (control 0 week: 26.4 ± 0.2 g, 1 week: 28.0 ± 0.2 g, 2 weeks: 28.2 ± 0.3 g, 3 weeks: 28.5 ± 0.6 g, 4 weeks: 28.4 ± 0.5 g; STZ 0 week: 26.5 ± 0.3 g, 1 week: 23.8 ± 0.7 g, 2 weeks: 22.6 ± 0.4 g, 3 weeks: 22.7 ± 0.7 g, 4 weeks: 22.1 ± 0.7 g; P < 0.01 control vs. STZ, n = 5). The rapid rise in blood glucose levels and the weight loss both suggest the onset of type 1 DM. These results indicate that STZ treatment causes type 1 DM at 1 week and that these mice are applicable as a type 1 diabetic mouse model (DM mice).

### GE acceleration in the early stages of DM

**Fig 2:**
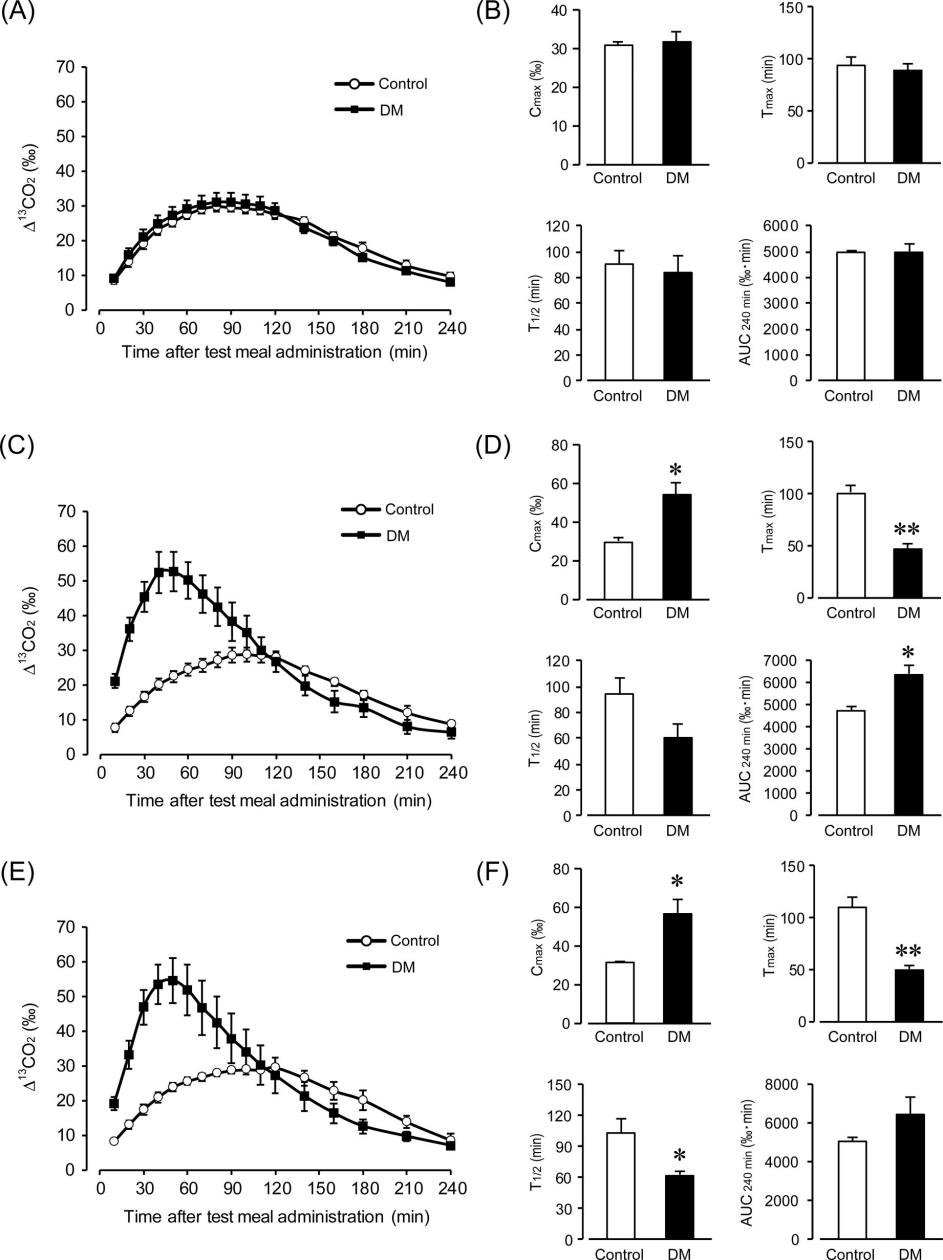
Accelerated gastric emptying in the early stage of type 1 diabetes. (A) and (B) Time-course of gastric emptying at 0 week following an injection of streptozotocin (STZ) in control and diabetic (DM) mice. Quantification of C_max_, T_max_, AUC_240min_, and T_1/2_ are calculated from (A). Each column shows the mean ± SEM (n = 5). (C) and (D) Time-course of gastric emptying, C_max_, T_max_, AUC_240min_, and T_1/2_ at 2 weeks following an injection of STZ in control and DM mice. Each column shows the mean ± SEM (n = 5). *P < 0.05, **P < 0.01; significantly different from control. (E) and (F) Time-course of gastric emptying, C_max_, T_max_, AUC_240min_, and T_1/2_ at 4 weeks following an injection of STZ in control and DM mice. Each column shows the mean ± SEM (n = 5). *P < 0.05, **P < 0.01; significantly different from control.

To investigate the changes in GE in the early stage of DM, we performed a ^13^C-octanoic acid breath test at 0, 2, and 4 weeks after STZ treatment. Fig 2A shows the ^13^CO_2_ excretion curve at 0 weeks (Fig 2A). In the control mice, the ^13^CO_2_ concentration slowly increased and showed a peak at 90–100 min and then decreased to near base value at 240 min. DM mice also showed the same curve as the control mice. Fig 2B shows C_max_, T_max_, AUC_240min_, and T_1/2_, which were calculated from the ^13^CO_2_ excretion curve (Fig 2B). As GE was accelerated, the values of C_max_ and AUC_240min_ increased, while T_max_ and T_1/2_ decreased. C_max_ and AUC_240min_ were not significantly different between control and DM mice (control C_max_: 30.6 ± 1.1 ‰, AUC_240min_: 4973 ± 72 ‰·min; DM C_max_: 31.7 ± 2.9 ‰, AUC_240min_: 4955 ± 325 ‰·min, n = 5). Similarly, T_max_ and T_1 / 2_ were not significantly different between control and DM mice (control T_max_: 94.0 ± 8.3 min, T_1/2_: 90.0 ± 11.5 min; DM T_max_: 88.7 ± 6.0 min, T_1/2_: 84.5 ± 11.9 min, n = 5). Fig 2C shows the ^13^CO_2_ excretion curve at 2 weeks (Fig 2C). In control mice, the ^13^CO_2_ concentration gradually rose from the start of the measurements. On the other hand, in DM mice, the ^13^CO_2_ concentration rapidly increased, and the disappearance of the ^13^CO_2_ concentration was rapid compared to that in the control mice. This result suggests an acceleration of GE. Fig 2D shows C_max_, T_max_, AUC_240min_, and T_1/2_ at 2 weeks (Fig 2D). C_max_ and AUC_240min_ were significantly increased in DM mice (control C_max_: 29.5 ± 1.8 ‰, AUC_240min_: 4675 ± 142 ‰·min; DM C_max_: 54.4 ± 5.8 ‰, AUC_240min_: 6289 ± 374 ‰·min; P < 0.05 control vs. DM, n = 5). In addition, T_max_ was significantly decreased in DM mice (control T_max_: 102.0 ± 5.2 min; DM T_max_: 48.0 ± 4.4 min; P < 0.01 control vs. DM, n = 5). T_1/2_ was similarly decreased in DM mice (control T_1/2_: 94.1 ± 12.7 min; DM T_1/2_: 60.2 ± 10.0 min, n = 5). Fig 2E shows the ^13^CO_2_ excretion curve at 4 weeks (Fig 2E). In the DM mice, ^13^CO_2_ concentration increased rapidly, and the disappearance of ^13^CO_2_ concentration was also rapid, which is similar to the result at 2 weeks. Fig 2F shows C_max_, T_max_, AUC_240min_, and T_1/2_ at 4 weeks (Fig 2F). C_max_ was significantly increased in DM mice (control C_max_: 40.0 ± 0.4 ‰; DM C_max_: 56.5 ± 6.7 ‰; P < 0.05 control vs. DM, n = 5). Similarly, AUC_240min_ was increased in DM mice compared to control mice (control AUC_240min_: 4993 ± 215 ‰·min; DM AUC_240min_: 6378 ± 868 ‰·min, n = 5). Additionally, T_max_ and T_1/2_ were significantly decreased in DM mice (control T_max_: 110.0 ± 9.8 min, T_1/2_: 101.5 ± 13.6 min; DM T_max_: 48.0 ± 4.4 min, T_1/2_: 59.0 ± 3.5 min; P < 0.01 control T_max_ vs. DM T_max_, P < 0.05 control T_1/2_ vs. DM T_1/2_, n = 5). These results indicate that GE is accelerated in the early stages of DM.

### GE in the early stage of DM was not affected by acute changes in blood glucose levels

**Fig 3:**
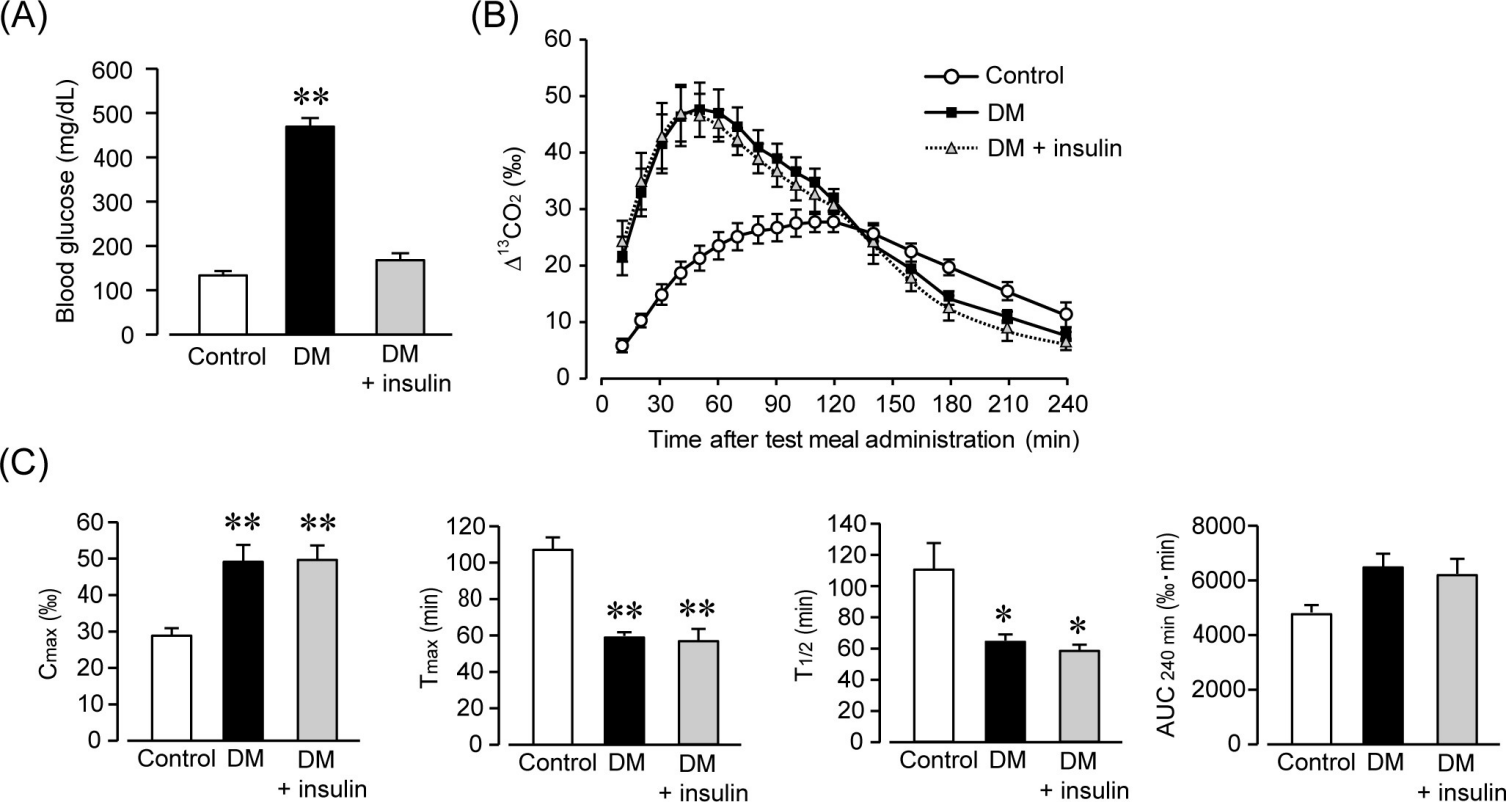
Accelerated gastric emptying in diabetic mice and treatment with insulin. (A) Blood glucose levels at 2–4 weeks following an injection of streptozotocin (STZ) in control, diabetic (DM), and treatment with insulin (DM + insulin) mice. Each column shows the mean ± SEM (n = 6). **P < 0.01; significantly different from control. (B) Time-course of gastric emptying measured by the ^13^C-octanoic acid breath test in control, DM, and DM + insulin mice. Each column shows the mean ± SEM (n = 6). (C) Quantification of C_max_, T_max_, AUC_240min_, and T_1/2_ are calculated from (B). Each column shows the mean ± SEM (n = 6). *P < 0.05, **P < 0.01; significantly different from control.

To determine whether accelerated GE is due to a temporary elevation in blood glucose levels at 2–4 weeks, we analyzed the GE function using the DM mice after a single administration of insulin. Fig 3A shows the blood glucose levels of each mouse (Fig 3A). The STZ treatment resulted in a significant elevation in blood glucose levels, and a single administration of insulin significantly reduced blood glucose levels to control levels (control: 132.5 ± 11.0 mg/dl; DM: 466.0 ± 18.3 mg/dl; DM + insulin: 166.1 ± 13.2 mg/dl; P < 0.01 control vs. DM, n = 6). Fig 3B shows the ^13^CO_2_ excretion curve in the ^13^C-octanoic acid breath test (Fig 3B). In control mice, the ^13^CO_2_ concentration gradually rose from the start of the measurements. On the other hand, in the DM and insulin administration groups, the ^13^CO_2_ concentration increased rapidly, and the disappearance of the ^13^CO_2_ concentration was also rapid. Fig 3C shows the results of the gastric emptying analysis using C_max_, T_max_, AUC_240min_, and T_1/2_ (Fig 3C). C_max_ was significantly increased in the DM and insulin administration groups (control C_max_: 28.6 ± 2.1 ‰; DM C_max_: 48.6 ± 4.7 ‰; DM + insulin C_max_: 49.1 ± 3.5 ‰; P < 0.01 vs. control, n = 6). AUC_240min_ was also increased in the DM and insulin administration groups in comparison to the control mice (control AUC_240min_: 4785 ± 269 ‰·min; DM AUC_240min_: 6483 ± 475 ‰·min; DM + insulin AUC_240min_: 6240 ± 508 ‰·min, n = 6). T_max_ and T_1/2_ were significantly decreased in the DM and insulin administration groups (control T_max_: 105.0 ± 7.4 min, T_1/2_: 108.8 ± 16.5 min; DM T_max_: 57.5 ± 2.3 min, T_1/2_: 62.3 ± 4.3 min; DM + insulin T_max_: 55.8 ± 5.6 min, T_1/2_: 58.4 ± 2.7 min; P < 0.01 and P < 0.05 respectively; vs. control, n = 6). These results suggest that accelerated GE in the early stages of DM is not associated with acute changes in blood glucose levels.

### Persistent hyperglycemia in the early stages of DM increased the networks of ICC

**Fig 4:**
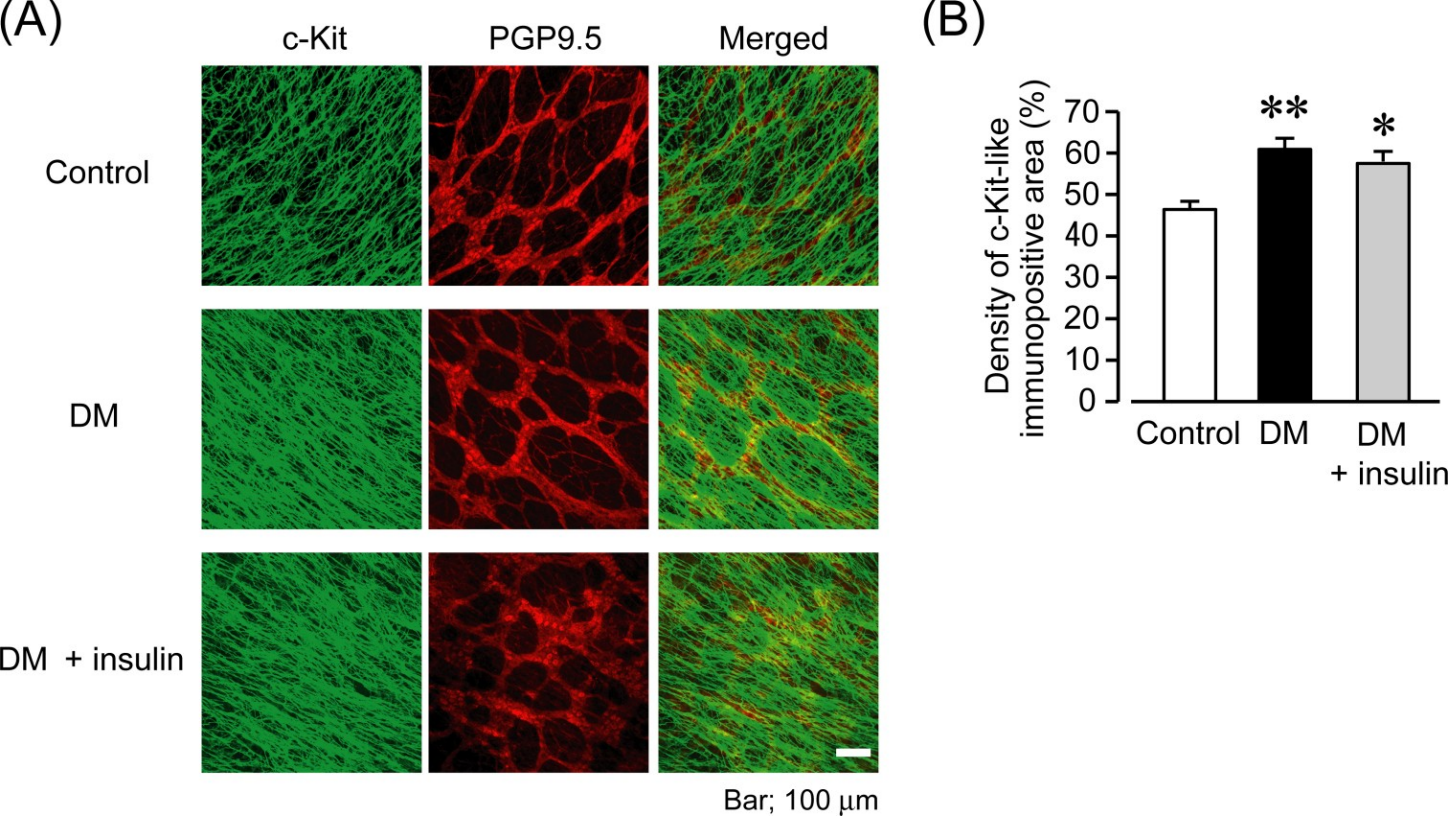
Hyperglycemia in the early stages of diabetes increases c-Kit-positive interstitial cells of Cajal. (A) Representative results of immunostaining for c-Kit (interstitial cells of Cajal marker; green) and PGP9.5 (neuron marker; red) in whole-mount preparations of gastric muscularis obtained from control, DM and DM + insulin mice at 2–4 weeks after STZ administration. Scale bar, 100 μm. (B) Quantification of the density of the c-Kit-positive area. Each column shows the mean ± SEM (n = 6, 5, 4). *P < 0.05, **P < 0.01; significantly different from control.

We investigated the changes in the networks of ICC in the early stages (2–4 weeks) of DM. Fig 4A shows the representative results of immunostaining for the gastric antrum (Fig 4A). In the present study, we used c-Kit and PGP 9.5 as markers for ICC and neurons, respectively. Fig 4B shows the quantified density of c-Kit-immunoreactive regions (Fig 4B). In the tissues from control mice, c-Kit-expressing ICC were localized at the level of ICC-MY and formed mesh-like networks. In the tissues from DM mice, c-Kit-immunoreactive ICC significantly increased when compared to control tissues (control: 46.2 ± 2.2%; DM: 60.9 ± 2.5%; P < 0.01 control vs. DM, n = 6, 5). Moreover, the tissues from DM mice with acute insulin treatment also showed remarkable increases in ICC networks; there was no significant difference between DM and DM + insulin mice. (DM + insulin: 57.3 ± 2.6%; P < 0.05 control vs. DM + insulin, n = 4). There were no significant differences in the structure of the enteric neurons between tissues from control, DM, and DM + insulin mice (data not shown). These results suggest that the hyperglycemic environment in the early stages of DM increases the networks of ICC, which may be responsible for accelerated GE.

### Diabetic mice in the early stages of DM have higher levels of oxidative stress

**Fig 5:**
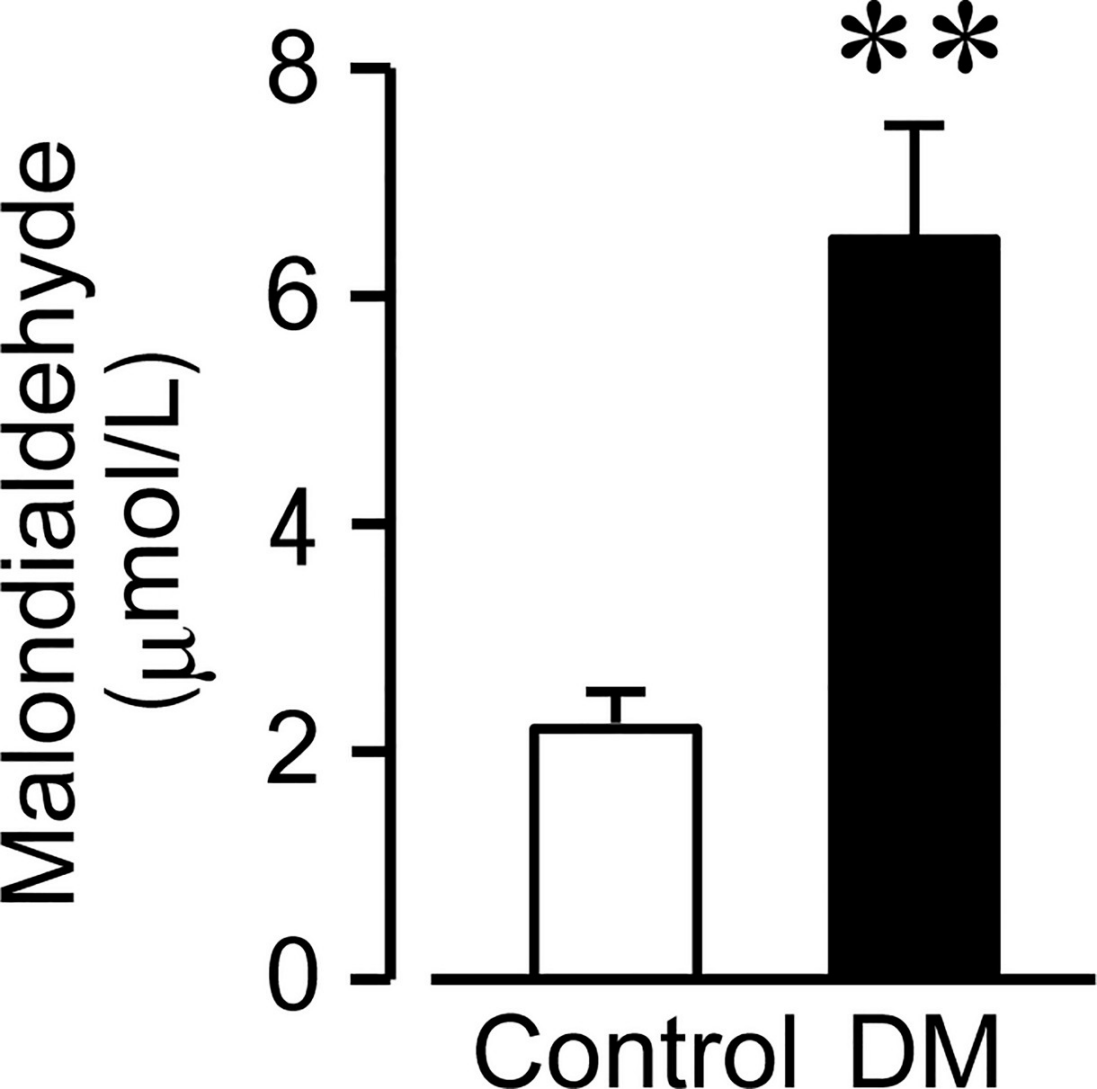
Oxidative stress levels at 2–4 weeks following an injection of streptozotocin in control and diabetic (DM) mice. Oxidative stress was determined by measuring serum thiobarbituric acid reactive substances as malondialdehyde, an indicator of lipid peroxidation. Each column shows the mean ± SEM (n = 6, 8). **P < 0.01; significantly different from control.

To investigate levels of oxidative stress in the early stage of DM, we measured serum TBARS as MDA, a product of polyunsaturated fatty acid peroxidation. Fig 5 shows the oxidative stress levels as MDA in control and DM mice (Fig 5). In DM mice, MDA significantly increased when compared to control mice (control: 2.2 ± 0.3 μmol/L; DM: 6.7 ± 1.0 μmol/L; P < 0.01 control vs. DM, n = 6, 8). These results suggest that hyperglycemia in the early stages of DM increased levels of oxidative stress.

## Discussion

In this study, we investigated GE and ICC networks in mice at the early stages of type 1 DM after induction by STZ. Our results showed accelerated GE and increased ICC networks in the early stages of DM. Moreover, these changes were not affected by the acute normalization of blood glucose levels.

In the present study, GE was accelerated 2 weeks after STZ administration. There is a possibility that the acceleration of GE at 2 weeks was caused by a toxic effect of STZ and not the DM condition. However, a similar pattern of accelerated GE at 1–2 weeks after the start of diabetic symptoms has been previously reported in spontaneous type 1 diabetic mice models [13]. It appears that accelerated GE is caused in the early stages of DM, which is in agreement with previous reports.

It has been reported in the literature that GE in diabetic mouse models is accelerated between 1–2 weeks, then returns to the normal GE pattern in 3–5 weeks and is delayed after the onset of DM for more than 1.5 months [13]. In this study, GE was not examined longer than 4 weeks after STZ administration. However, our results are consistent with a previous report in which GE was accelerated in the early stages of DM, although there is a difference in the time course. On the other hand, in the case of human patients with type 1 and type 2 DM, accelerated GE has been reported to occur regardless of the timing of the onset of DM [2]. This difference may indicate that the period after DM onset is not the only factor accelerating GE. Further investigations are needed to clarify what kind of factors decides GE functioning. The analysis of mice at the early stages of DM as a model for accelerated GE in DM may answer this question.

Accelerated GE is seen in patients and rodents with both type 1 and type 2 DM [2,18]. Hyperinsulinemia is the only feature of type 2 DM, but not type 1 DM. Therefore, hyperglycemia is the more likely factor leading to changes in GE function. In the present study, we acutely normalized the blood glucose concentration of DM mice after the establishment of accelerated GE by single dose of insulin. However, insulin administration failed to recover normal GE. This result suggests that hyperglycemia accelerated GE not due to acute changes in cell functions but due to organic changes in GI networks composed of interstitial cells, such as smooth muscle cells, neurons, and ICC. Indeed, we found that ICC networks significantly increased in tissues from DM mice both with and without a single dose of insulin.

Previous study have reported that GE is accelerated in mice models with genetic ICC hyperplasia [19]. Moreover, type 2 diabetic mice with increased ICC networks show significant increases in the frequency of slow waves and contractions in response to cholinergic stimulation [3]. This evidence suggests that accelerated GE in the early stages of DM is caused by increased ICC networks. On the other hand, GI motility is produced not only by ICC but also by neurons, smooth muscle cells, and platelet-derived growth factor receptor-α positive interstitial cells (PDGFRα^+^ cells). In this study, there were no significant differences in the structure of the enteric neurons between control and DM mice. In general, it is thought that the damage of the enteric nerve is caused by the blood flow disorder and the accumulation of sorbitol due to the chronic course of hyperglycemic condition. Therefore, progression of the disease stage in diabetes can lead to loss of the enteric nerve. In the present study, mice in the early stages of DM (2–4 weeks) were used, and it is possible that hyperglycemia did not have enough time to damage the enteric nerve. On the other hand, there is a report stating that the release of inhibitory neurotransmitters from neurons was significantly suppressed in the gastric antrum during the early stage of hyperglycemia in NOD mice, a type 1 DM model [20]. Thus, in addition to ICC, there is a need to investigate the morphology and function of smooth muscle cells, neurons, and PDGFRα^+^ cells to clarify the mechanisms behind accelerated GE in the early stage of DM.

Persistent hyperglycemia increases superoxides through the activation of NADPH oxidase (Nox) and metabolism in the mitochondrial electron transport system. Previous reports have demonstrated that the downregulation of heme oxygenase-1, an antioxidant enzyme expressed in resident macrophages, causes ICC loss and dysfunction in DM [21,22]. Another report has also suggested that the maintenance of ICC networks in DM is dependent on the ability of anti-inflammatory macrophages to suppress the high levels of oxidative stress [10]. These findings indicate that decreases in the ICC networks in DM are likely to be dependent on oxidative stress. On the other hand, the mechanism behind the increase in the ICC networks is not well understood. Ets variant 1 (ETV1) is a transcription factor that can upregulate the expression of c-Kit. It has been reported that a gastrointestinal stromal tumor arising from ICC shows high expression levels of c-Kit through the stabilization of ETV1, which results in an acceleration of tumor growth [23–25]. A recent report has indicated that the oxidative metabolism of glucose induces activation of mitogen activated protein kinase 1 (MAPK1) and MAPK3, leading to c-Kit upregulation via the stabilization of ETV1 [3]. This finding implies that hyperglycemia increases ICC through oxidative stress, leading to accelerated GE. Thus, hyperglycemia-related oxidative stress is suggested to cause both a decrease and an increase in ICC, which results in delayed and accelerated GE, respectively. The fate of ICC in hyperglycemia seems to be determined by the degree of oxidative stress. In the present study, we measured serum MDA and determined oxidative stress levels in the early stages of DM. In fact, MDA significantly increased in DM mice. This result suggests that in cases of early stage DM, hyperglycemia-produced oxidative stress may be insufficient to promote the loss of ICC networks. It can be suggested that accelerated GE, as observed in the early stage of DM, may occur when the DM-related factors that support ICC networks outweigh the effects of adverse factors. Factors of accelerated GE may include Nox activation, glycation, and oxidative stress derived from glucose metabolism in the mitochondrial electron transport system.

In conclusion, our results provide the first evidence that accelerated GE has already occurred in the early stages of DM and that it is accompanied by increased networks of ICC in the gastric antrum. The increase in ICC networks observed in the early stages of DM is a key determinant of accelerated GE. This mechanism may help to identify the diabetic factor that correlates with the onset of DM and GE disorder.

## Supporting information

S1 Dataset Minimal data set. (ZIP)
